# Benefits of Cardamom (*Elettaria cardamomum* (L.) Maton) and Turmeric (*Curcuma longa* L.) Extracts for Their Applications as Natural Anti-Inflammatory Adjuvants

**DOI:** 10.3390/plants10091908

**Published:** 2021-09-14

**Authors:** Gustavo R. Cárdenas Garza, Joel H. Elizondo Luévano, Aldo F. Bazaldúa Rodríguez, Abelardo Chávez Montes, Raymundo A. Pérez Hernández, Ameyalli J. Martínez Delgado, Sonia M. López Villarreal, José Rodríguez Rodríguez, Rosa M. Sánchez Casas, Uziel Castillo Velázquez, Osvelia E. Rodríguez Luis

**Affiliations:** 1 Faculty of Dentistry, Autonomous University of Nuevo León, Monterrey 64460, NL, Mexico; gustavo.cardenasgrz@uanl.edu.mx (G.R.C.G.); raymundo.perezhrz@uanl.edu.mx (R.A.P.H.); ameyalli.martinezd@uanl.edu.mx (A.J.M.D.); sonia.lopezvl@uanl.edu.mx (S.M.L.V.); 2 Faculty of Biological Sciences, Autonomous University of Nuevo León, San Nicolás de los Garza 66455, NL, Mexico; joel.elizondolv@uanl.edu.mx (J.H.E.L.); aldo.bazalduarg@uanl.edu.mx (A.F.B.R.); abelardo.chavezmn@uanl.edu.mx (A.C.M.); 3 Tecnológico de Monterrey, Monterrey 64849, NL, Mexico; jrr@tec.mx; 4 Faculty of Veterinary Medicine and Zootechny, Autonomous University of Nuevo León, Monterrey 64460, NL, Mexico; rosa.sanchezcss@uanl.edu.mx

**Keywords:** anti-inflammatory, *Curcuma longa*, cytokines, cytotoxic activity, *Elettaria cardamomum*, extracts, inflammation, medicinal plants, natural products, phytochemicals

## Abstract

The genus *Zingiberaceae* has been widely used for phytotherapeutic purposes in traditional medicine throughout the world for its anti-inflammatory activity. Experimental studies have established that inflammation caused by chronic infections represents a risk factor for different forms of cancer. The objective of this study was focused on determining the anti-inflammatory capacity and cytotoxic activity of aqueous extracts of *Elettaria cardamomum* (cardamom) and *Curcuma Longa* (turmeric). The extracts were obtained by maceration and, through GC-MS/MS, a total of 11 different chemical components were determined in the aqueous extract of cardamom and 7 in the extract of turmeric. The main compounds found in cardamom and turmeric were *α*-terpinyl acetate (54.46%) and *β*-turmerone (33.45%), respectively. RT-qPCR results showed significantly lower gene expression levels of innate inflammatory cytokines (IL-6 and TNF-*α*) compared to the control (LPS). Also, it was observed that the extracts do not possess cytotoxic activity against different cell lines, where *E. cardamomum* showed EC_50_ (µg/mL) of 473.84 (HeLa cells), 237.36 (J774A.1 cells), 257.51 (Vero E6 cells), and 431.16 (Balb/C peritoneal cells) and *C. longa* showed EC_50_ (µg/mL) of 351.17 (HeLa cells), 430.96 (J774A.1 cells), 396.24 (Vero E6 cells), and 362.86 (Balb/C peritoneal cells). The results of this research suggest that natural extracts of *E. cardamomum* and *C. longa* possess anti-inflammatory effects and no cytotoxic activity against HeLa, J774A.1, Vero E6, and Balb/C peritoneal cell lines. Finally, it was observed that the extracts also decreased nitric oxide (NO) production in peritoneal macrophages.

## 1. Introduction

Nowadays, there is a tendency to incorporate phytotherapy as an efficient therapeutic option worldwide; the use of medicinal plants to treat various pathologies has been reported since they possess active principles with different biological actions [1,2]. The World Health Organization (WHO) encourages the use of plants with a scientific basis for the therapy of systemic diseases [3]. These diseases can lead to serious complications, activating the inflammation process with infiltration of macrophages, neutrophils, lymphocytes, and plasma cells in the tissue, with the release of cytokines that contribute to the repair of tissue damage [4]. The inflammation process occurs as a response to aggression to repair and restore tissues [5]. During this period, cytokines, proteins that regulate the function of immune system cells produced by lymphocytes, macrophages, or monocytes and hematopoietic cells with pro-inflammatory or anti-inflammatory action, are released [6]. Among the proinflammatory cytokines are tumor necrosis factor-alpha (TNF-*α*), interleukin-1*α* (IL-1*α*), interleukin-1*β* (IL-1*β*), interleukin-6 (IL-6), interleukin-17 (IL-17), and, with antagonistic effects, interleukin-10 (IL-10) [7,8]. Current therapy involves anti-inflammatory agents that, in some cases, may reveal adverse effects such as the presence of a peptic ulcer, gastrointestinal toxicity, myocardial infarction, heart failure, stroke, or nephrotoxicity [9,10].

In traditional medicine, the antioxidant and pharmaceutical properties of some plants belonging to the *Zingiberaceae* family, such as *Elettaria cardamomum* and *Curcuma longa*, have been well studied, and have found applications in industries such as food, cosmetics, and pharmaceuticals, among others; these capabilities are related to their diverse composition of various phytochemical compounds, mainly phenolic compounds [11]. *E. cardamomum* (cardamom), also known as “green or true cardamom”, is a perennial herbaceous plant, which has been described as possessing compounds such as phenols, starch, tannins, terpenoids, flavonoids, proteins, sterols [12], anthocyanins, and alkaloids, and it also possesses various pharmacological properties, such as antioxidant, anti-inflammatory, anticancer, and antimicrobial activities [13]. *C. longa*, also called turmeric or curcuma, possesses different bioactive components such as curcuminoids (curcumin) and essential oils (monoterpenes) [14], and among its multiple therapeutic properties are anti-inflammatory, antihyperlipidemic, antimicrobial, and antiparasitic activity [15,16]. Its traditional use has been reported for the treatment of diseases such as rheumatoid arthritis, multiple sclerosis, and psoriasis as it manages to modulate the signaling of proinflammatory cytokines [17].

The main objective of this research focused on the evaluation of the effects of crude aqueous extracts of *E. cardamomum* and *C. longa* on the inflammatory process through the expression of interleukins by stimulated macrophages and their possible applications as adjuvants in health improvement therapies.

## 2. Materials and Methods

### 2.1. Chemicals and Reagents

All chemicals and solvents were analytical grade. Antibiotic antimycotic solution (100×) stabilized, diethyl polycarbonate (DEPC), dimethyl sulfoxide (DMSO), Dulbecco’s Modified Eagle Medium (DMEM), fetal bovine serum (FBS), glycine buffer solution, Griess reagent, guanidine thiocyanate, lipopolysaccharide (LPS) from *Escherichia coli* O26:B6 smooth strain, n-hexane, nystatin, RNAzol^®^ RT, RPMI-1640 medium with L-glutamine, SYBR^®^ Green Quantitative RT-qPCR kit, and thiazolyl blue tetrazolium bromide (MTT) were purchased from Sigma-Aldrich (Merck KGaA, Darmstadt, Germany). GoTaq^®^ qPCR Master Mix kit and ImProm-II™ Reverse Transcription System reverse transcription kit was purchased from Promega (Promega^®^ Corporation, Madison, WI, USA).

### 2.2. Plant Material and Extraction

*E. cardamomum* seeds (SKU: 209740-01) and *C. longa* rhizome (SKU: 205400-54) were both purchased from Starwest Botanicals (Sacramento, CA, USA). Plant material was purchased in powdered form. The extraction was performed with distilled water. Aqueous extracts of both plants were obtained by the heat infusion method (100 °C/100 rpm) for 30 min. For this purpose, 10 g of plant material were placed in 100 mL of distilled water in a flat-bottomed ball flask. After the extraction time, the extracts were filtered, frozen at −80 °C, and, finally, lyophilized. The following formula was used to calculate the extraction yield percentage [18]:

(1) $$\%\;\mathrm{Yield}=\left(\frac{\mathrm{Final}\;\mathrm{weight}}{\mathrm{Initial}\;\mathrm{weight}}\right)\times 100$$

### 2.3. Qualitative Phytochemical Tests

The following phytochemical tests were performed to identify the functional groups of each extract: Lieberman-Buchard (sterols, triterpenes), Shinoda (flavonoids), Baljet (sesquiterpene lactones), sulfuric acid (quinones), and ferric chloride (tannins) [19], then each extract was analyzed by gas chromatography coupled to GC/MS mass for identification of the main components of its chemical composition [11].

### 2.4. GC and GC-MS/MS Analysis of the Aqueous Extracts

For chemical identification and quantification, 1 μL of diluted aqueous extract in n-hexane (1:20, *v*:*v*) was analyzed using a gas chromatograph Varian Saturn 2100T coupled to an MS/MS Saturn 2100 ion trap mass spectrometer (Agilent Technologies, Inc., Walnut Creek, CA, USA). The chromatographic separation was performed with the capillary column HP-5ms (30 m × 0.25 mm × 0.25 μm). The split injection mode was used (split ratio 1:20), while helium-6 was used as the carrier gas with a flow rate of 1 mL min^−1^. The injector temperature was set to 250 °C. The initial oven temperature was set to 70 °C for 1 min, then the temperature was raised to 150 °C at a rate of 5 °C per min and held at this temperature for 5 min. In addition, the column was heated up to 200 °C at a rate of 10 °C and, finally, kept at 200 °C for 15 min. The total running time was 60 min. The mass spectra were recorded in the SCAN mode in a range from 30 to 400 *m*/*z* using electron ionization energy at 70 eV and the detector temperature was set to 150 °C [11]. The GC-MS structure analysis was performed using the National Institute Standard and Technology (NIST) database; in addition, the results were compared with results from previous studies available in the literature [20,21].

### 2.5. Gene Expression of Anti-Inflammatory Cytokines on Peritoneal Macrophages Stimulated with Aqueous Extracts and Challenged with Lipopolysaccharide

To evaluate the anti-inflammatory effects of aqueous extracts of *E. cardamomum* and *C. longa*, murine macrophages were challenged with lipopolysaccharide (LPS; Escherichia coli serotype O26:B6 smooth strain) to induce a classical activation phenotype [22]. Relative quantification of proinflammatory cytokine gene sequences IL-4, IL-6, IL-10, and TNF-α induced by aqueous extracts of *E. cardamomum* and *C. longa*, as well as LPS, were measured by real-time quantitative PCR (RT-qPCR) using SYBR^®^ Green reagent.

#### 2.5.1. Obtaining Peritoneal Cells

Five female Balb/C mice were inoculated intraperitoneally (IP) with 1 mL of DMEM, then sacrificed by cervical dislocation, and a gentle massage of the abdomen was performed. The culture medium with the cells in the peritoneal fluid was extracted and centrifuged at 2700 rpm (4 °C) for ten minutes, eliminating the supernatant and conserving the cell pellet, which was homogenized with the aqueous extract individually (Table 1). Finally, it was incubated for 30 min at 37 °C (CO_2_ 5%), centrifuged, and the supernatant was eliminated [23]. Two washes were performed with phosphate buffer (PBS, ph 7.4) and then homogenized with 500 μL of RNAzol^®^. As an inducer of inflammation in macrophages, 50 µg/mL of LPS was used as a positive control [24]. The concentrations of the extracts were obtained from microbicide assays in which these concentrations were used (data not shown).

#### 2.5.2. RNA Extraction

Extraction of total ribonucleic acid (RNA) from peritoneal macrophages was performed from samples suspended in the cell lysis reagent RNAzol^®^, guanidine thiocyanate. Subsequently, total RNA extraction was performed according to the manufacturer’s instructions. Once the total RNA pellet was obtained, it was suspended in 20 μL of nuclease-free water obtained by DEPC treatment. The total RNA pellet obtained was quantified in a NanoDrop™ ND-1000 spectrophotometer (Thermo Fisher Scientific Inc., Wilmington, DE, USA) at optical densities (OD) of 260 and 280 nm; for this purpose, total RNA was suspended in 20 μL of nuclease-free water. The extraction purity and concentration showed a variation of 1.98 and 2.09 and a concentration ranging from 485.45 to 940.39 ng/μL for each of the triplicates [24].

#### 2.5.3. Complementary DNA (cDNA) Synthesis

From the previously obtained total RNA, complementary DNA (cDNA) synthesis was performed using the ImProm-II™ Reverse Transcription System reverse transcription kit. For each reaction, 4 μL of ImProm-II™ 5X reaction buffer, 1 μL of the dNTP’s mixture (0.5 mM of each), 2.4 μL of MgCl_2_ (25 mM), 1 μL of Oligo(dT)_15_ primer, 1 μL of ImProm-II™ transcriptase, 1500 ng of RNA quencher, and nuclease-free water were mixed to a final volume of 20 μL. The reactions were carried out in 200 μL capacity microtubes. The prepared samples were incubated in a thermal cycler (Veriti^®^, Applied Biosystems^®^, Waltham, MA, USA) to carry out cDNA synthesis, the reaction conditions used were as follows: 25 °C (5 min), 42 °C (60 min), 70 °C (15 min), and 4 °C (∞). Once the retrotranscription was completed, the concentration and purity of the cDNA obtained were determined using an EPOCH™ microplate spectrophotometer (BioTek Instruments, Inc., Winooski, VT, USA) with the Gen5 software for microplate reading and data analysis. Finally, the products obtained were stored at −80 °C until further use.

#### 2.5.4. Oligonucleotide Design

The design of oligonucleotides for the quantification of cytokines (IL-4, IL-6, IL-10, and TNF-*α*) and endogenous genes (*β*-actin and Gpd-1) was performed from the mRNA sequence of each gene, obtained from Genbank. (Table 2). The oligonucleotides for the amplification of the eight selected sequences were designed using the Primer Quest™ Tool online program from IDT™ (Integrated DNA Technologies, Inc., Coralville, IA, USA), which was responsible for performing the synthesis of the oligonucleotides used in this project. The oligonucleotides were designed according to the following specifications: approximate size of 20 bp, melting temperature of 60 °C, and a guanine-cytokine content of 55%. The oligonucleotides obtained were analyzed using the PRIMER BLAST program to verify their specificity with the sequence of interest.

#### 2.5.5. Real Time—qPCR (RT-qPCR)

Quantification of gene expression by RT-qPCR was calculated using the 2^−ΔΔCt^ method, which consisted of subtracting the threshold cycle (Ct) of the endogenous gene (Gpd1) from the Ct of the gene of interest, thus obtaining the ΔCt, then the average ΔCt of the control group was obtained and this was subtracted from the ΔCt of each of the biological samples, thus calculating the ΔΔCt, and, finally, the formula 2^−ΔΔCt^ was applied. The Ct was determined based on the default baseline assigned by the system [25]. The amplification reaction was performed using the GoTaq^®^ qPCR Master Mix kit at a final volume of 20 μL for each sample, including 10 μL of the GoTaq qPCR Mix (2x), 1 μL of the oligonucleotide pair of interest that (100 μM), 1 μL of the cDNA of interest (75 ng), and, finally, the reaction was completed with 8 μL of nuclease-free water. The qPCR reactions were carried out in a 96-well PCR microplate (Axygen Scientific^®^, Union City, CA, USA) coated with Platemax^®^ UltraClear Sealing Film (Axygen Scientific^®^, Union City, CA, USA). Each sample was placed in triplicate in the plate and, in addition, a control reaction was included, which consisted of adding all the reagents used to perform the amplification reaction except cDNA.

### 2.6. Index of Cytotoxicity of the Aqueous Extract of E. cardamomum and C. longa on Cell Cultures

For the evaluation of cytotoxicity of the aqueous extracts of *E. cardamomum* and *C. longa*, three cell lines were used, cervical cancer (HeLa, ATCC CCL-2), mouse macrophages (J774A.1, ATCC TIB-67), and African green monkey kidney (Vero E6, ATCC CRL-1586). All cells were maintained in RPMI-1640 medium, which contained L-glutamine, 10% FBS, and antibiotic/antifungal (penicillin, streptomycin, and amphotericin B), and were kept in incubation at 37 °C (5% CO_2_) in Corning^®^ 25 cm^2^ cell culture flasks (Merck KGaA, Darmstadt, Germany). Medium changes were carried out every third day. Passages were made when the cells reached approximately 80% confluence. Next, 100 μL of culture medium containing 5 × 10^4^ cells/well of each cell line were placed in a 96-well microplate and the different serial concentrations of the aqueous extract of *E. cardamomum* and *C. longa* were added, ranging from 3.125 to 200 μg/mL (200 μL final volume), which were then incubated for 24 h. A positive control (nystatin 100,000 μL/mL) and negative control (cells without treatment) were also included. Once the treatment time with the extracts was fulfilled, the MTT colorimetric test was carried out [23]. The culture medium was extracted and, subsequently, the plates were washed with PBS and 100 μL of tetrazolium salt MTT was added in 0.25 mg/mL to the non-supplemented medium. The plates were incubated at 37 °C/4 h. After that time, the supernatant was removed and 100 µL of DMSO + 20 μL of glycine buffer was added and incubated for 30 min to allow the formazan crystals to dissolve [26]. Subsequently, the absorbance was read on an EPOCH™ microplate reader at 570 nm and was analyzed with Gen5 software. Similarly, the same tests were performed on mouse (Balb/C) peritoneal macrophages to observe changes or differences that could be found between commercial and ex vivo cells. Percentage of cytotoxicity was calculated according to Equation (2) and the IC_50_ was determined [18].

(2) $$\%\;\mathrm{Cytotoxicity}=\left(\frac{\mathrm{Abs}\;\mathrm{of}\;\mathrm{the}\;\mathrm{sample}}{\mathrm{Abs}\;\mathrm{control}}\right)\times 100$$

### 2.7. Nitric Oxide Assay

To perform the nitric oxide (NO) assay, peritoneal cells were cultured for 22 h with 100 µg/mL of aqueous extract of *E. cardamomum* and 70 µg/mL of aqueous extract of *C. longa*. As an inflammation model, 50 ng/mL of LPS from *E. coli* O26:B6 smooth strain was used to induce a classical activation phenotype associated with an inflammatory process by NO production, determined by nitrite accumulation in the supernatant using Griess reagent [27].

### 2.8. Statistical Analysis

All results were expressed as mean value ± standard deviation. Each experiment was performed in triplicate independently in at least three replicates. The IBM-SPSS software (Ver. 22, IBM Corp., Armonk, NY, USA) was used for the statistical evaluation of the results obtained. For that purpose, a one-way analysis of variance (ANOVA) at a 95% confidence level and a Tukey post hoc test was applied. GraphPad Prism 6 was used to generate the graphs. The half-maximal effective concentration (EC_50_) was determined by the Probit test.

## 3. Results

### 3.1. Phytochemical Tests

The aqueous extract of *E. cardamomum* was positive for sterols, triterpenes, flavonoids, sesquiterpene lactones, and tannins. The extract of *C. longa* was positive for sterols, triterpenes, flavonoids, and sesquiterpene lactones (Table 3). Both extracts were negative for quinones. The extraction yields were 5.64% and 9.83%, respectively.

### 3.2. Compound Identification

**Figure 1:**
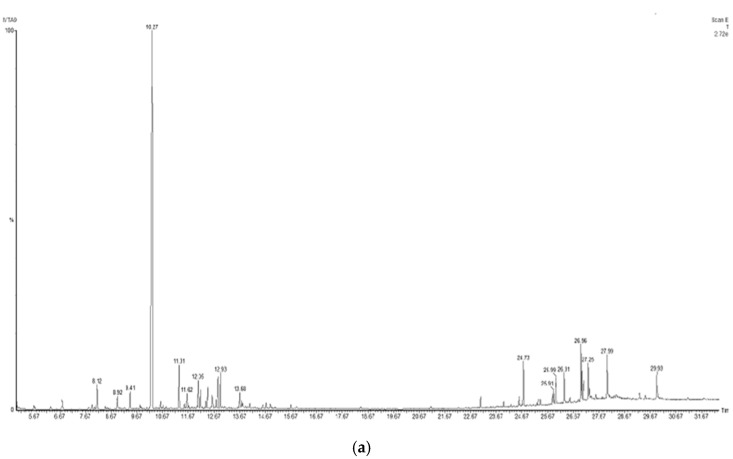

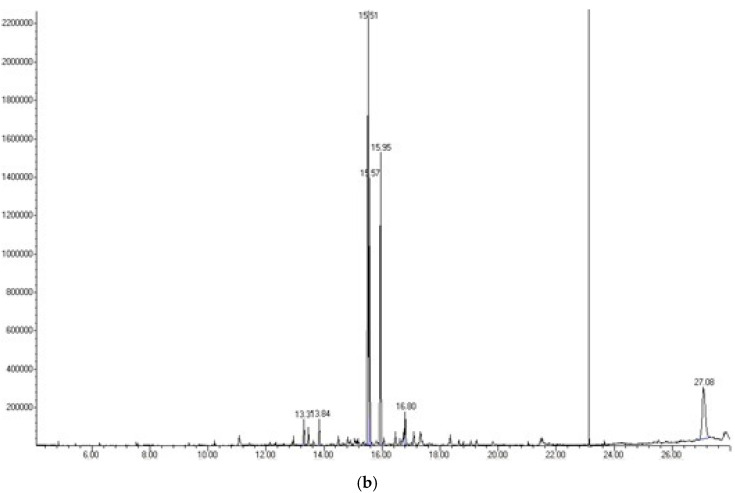
Chromatograms obtained from GC-MS screening of the aqueous extracts of *E. cadamomum* seeds (**a**), and *C. longa* roots (**b**).

To identify the compounds of interest, GC-MS/MS-based phytochemical analysis of the aqueous extracts was performed (Table 4); the analyses resulted in the identification of 11 major compounds in *E. cardamomum*, the α-terpinyl acetate, 2-((1R,4R)-4-hydroxy-4-methylcyclohex-2-enyl) propan-2-yl acetate, 9-hexacosene, heneicosane, 8-acetoxycarvotanacetone, geranyl oleate, *γ*-sitosterol, naphthalene, decahydro-4a-methyl-1-methylene-7-(1-methylethenyl)-,[4aR-(4a*α*,7*α*,8a*α*)], α-terpineol, (Z)-3,7-dimethylocta-2,6-dien-1-yl palmitate, and pentacosane (Figure 1a). In *C. longa*, *β*-turmerone, *α*-turmerone, Ar-turmerone, 16-kauren-19-yl acetate, *α*-atlantone, *β*-sesquiphellandrene, and benzene were identified (Figure 1b). The compounds identified in both aqueous extracts represent the bioactive compounds previously reported for both plants [11].

### 3.3. Gene Expression of Anti-Inflammatory Cytokines

The gene expression levels of the anti-inflammatory cytokines, Il-4 (*p* < 0.01) and Il-10 (*p* < 0.001), were analyzed to evaluate whether the aqueous extracts have an immunomodulatory effect on the expression of cytokines that regulate or promote inflammatory processes in peritoneal macrophages by using LPS as inflammation control (Table 5).

**Figure 2:**
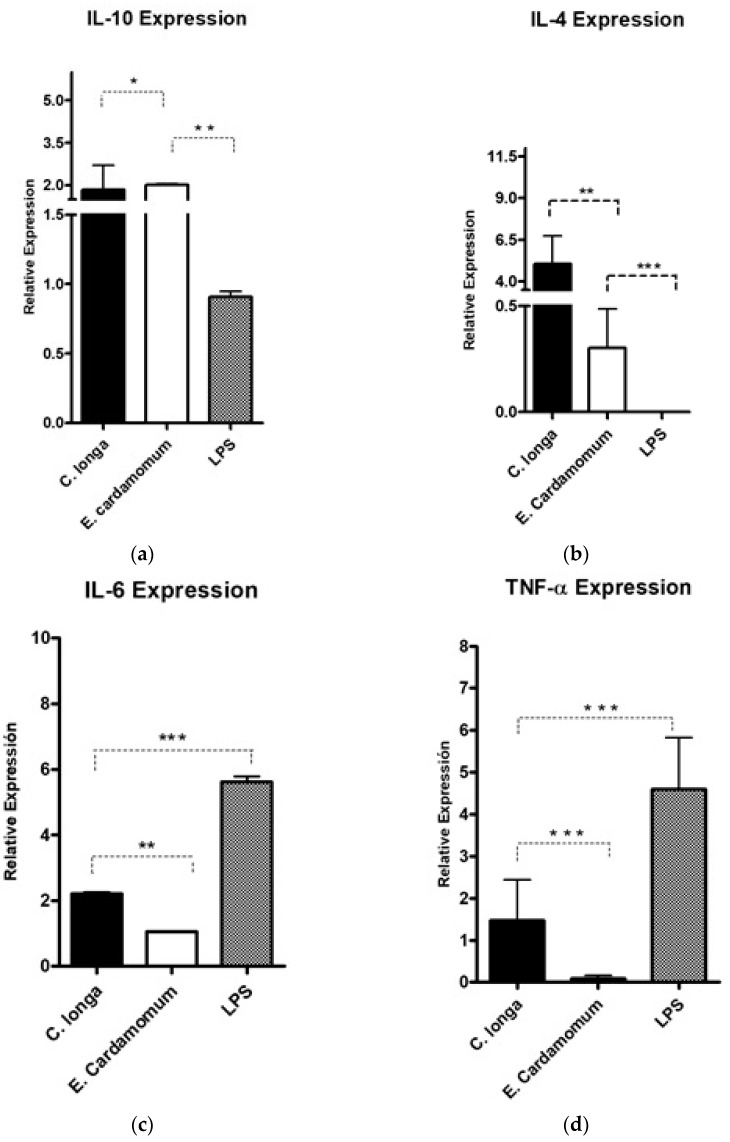
Gene expression of pro-inflammatory cytokines IL-10 (**a**), IL-4 (**b**) IL-6 (**c**), and TNF*-α* (**d**) in peritoneal macrophages stimulated by aqueous extracts of *E. cardamomum* and *C. longa*, challenged with LPS. * *p* < 0.05, ** *p* < 0.01, *** *p* < 0.001.

A significant increase in IL-10 gene expression level (Figure 2a) was observed with *C. longa* extract compared to *E. cardamomum* (*p* ≤ 0.05), as well as the same degree of significance compared to LPS (*p* ≤ 0.01); however, when IL-4 gene expression was analyzed, highly significant differences were observed between both aqueous extracts (*p* < 0.01) and highly significant differences (*p* ≤ 0.001) were also observed between the LPS control and both extracts (Figure 2b). These results suggest that after induction with LPS, the extracts exhibited a decrease in the inflammatory process produced by bacterial endotoxins (LPS), therefore, we can consider those extracts as modulators in inflammatory processes in the presence of Gram-negative microorganisms.

Highly significant (*p* < 0.001) decreases in the gene expression levels of proinflammatory cytokines IL-6 (Figure 2c) induced by aqueous extracts of both plants compared to LPS were observed (Table 5). Similar results were obtained for TNF*-α* (Figure 2d), which is a potent proinflammatory interleukin, observing highly significant decreases (*p* ≤ 0.001) for the treatments of each of the extracts with respect to the LPS control. The results presented in Figure 2 and Table 5 reveal that the extracts evaluated decreased the gene expression of proinflammatory cytokines, showing differences in expression in relation to that produced by LPS, which makes them potential natural active ingredients to reduce inflammatory processes.

### 3.4. Cytotoxic Activity of the Aqueous Extract

For the evaluation of the cytotoxicity of aqueous extracts of *E. cardamomum* and *C. longa*, three cell lines, HeLa, J774A.1, and Vero E6, and peritoneal cells of female Balb/C mice were used (Table 6). A dose–response relationship was observed, as cytotoxicity enhanced as the concentrations of the extracts increased. However, no high activity was observed against the cell lines or in peritoneal cells at the concentrations tested; *E. cardamomum* presented EC_50_ higher than 321.20 µg/mL on the three cell lines tested, while the EC_50_ of *C. longa* was higher than 190.79 µg/mL. *E. cardamomum* was the most active on J774A.1 (EC_50_ 237.36 µg/mL) and Vero E6 (EC_50_ 257.51 µg/mL), while *C. longa* was more effective on HeLa (EC_50_ 351.17 µg/mL). No differences were observed between the activities of the aqueous extracts when evaluated in peritoneal macrophages.

### 3.5. Nitric Oxide Depletion in Peritoneal Macrophages

**Figure 3:**
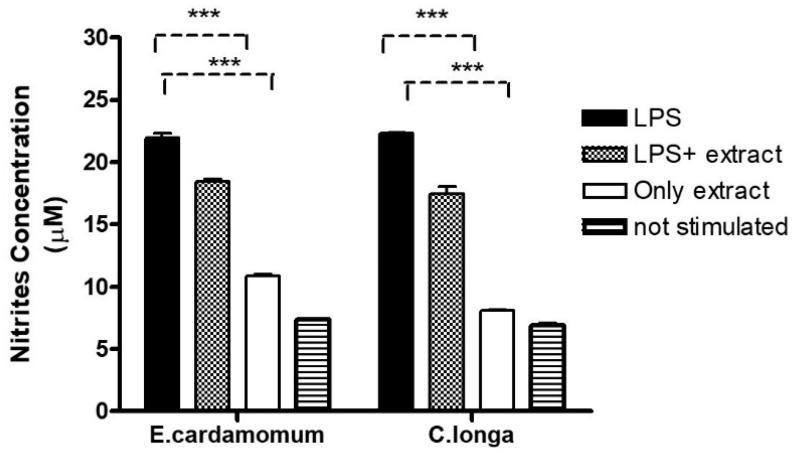
Nitric oxide production in peritoneal macrophages stimulated with 100 µg/mL of aqueous extracts of *E. cardamomum*, and 70 µg/mL of *C. longa*, challenged with 50 µg/mL of LPS. *** *p* < 0.001.

NO production was measured as an indicator of inflammation in peritoneal macrophages. Nitric oxide production averaged 22.12, 18.64, 10.91, and 7.45 µM nitrite for macrophages stimulated with LPS, LPS plus *E. cardamomum* extract, *E. cardamomum* extract alone, and without stimulation, respectively, while for *C. longa*, the results were 22.27, 16.97, 8.03, and 6.73 µM nitrite under the same treatments, showing significant differences (*p* < 0.001) when macrophages were incubated with 50 ng/mL LPS to induce an activation state (Figure 3).

## 4. Discussion

The use and evaluation of the traditional folk plants *E. cardamomum* and *C. longa* in terms of their anti-inflammatory activities have been remarkable because of the positive results obtained in several studies where these plants have been used to reduce the symptoms of various chronic inflammatory diseases [28]. In the development of such research, numerous bioactive compounds present in its essential oils have been identified, which differ in proportions and presence depending on the region where the plant was collected, the time of collection, or the type of extraction [13]. In the chemical characterization of *E. cardamomum* and *C. longa*, a series of major compounds were identified in the crude extracts of both plants, which largely comprise the composition of their essential oils, showing their participation in the immune response by acting as mediators in the synthesis of key factors of the immune response [29].

In the present investigation, the compounds identified as major in *E. cardamomum* have been previously reported as components of its essential oils [30]. We determined the anti-inflammatory activity of the aqueous extract of this plant, which coincides with previous reports [13], and while this activity is justified by the compound 1,8-cineole, this was not one of the majority compounds of the crude extract obtained by us. Instead, the combined activity of the compounds 9-hexacosene, *α*-terpinol, and linalool represents an alternative option for bioactive compounds with anti-inflammatory activity derived from this plant. Previous reports demonstrated the activity of 9-hexacosene in reducing the size of edema in mouse ears, induced by dimethylbenzene [31]; on the other hand, the ability of *α*-terpinol to suppress pro-inflammatory mediators generating an inhibition of IL-6 has been identified [32]. Similarly, in previous works, it was reported that linalool generates a reduction of TNF-*α* in addition to an inhibition of neutrophil activation [33]. Regarding the compound *α*-terpenyl acetate, the major compound in our *E. cardamomum* extract, it has not been previously associated with anti-inflammatory activity. Although this compound has been previously reported in plants with anti-inflammatory activity [13,34,35,36], including the plant under study, there is a lack of evaluations demonstrating such activity, which represents an opportunity to consider for future research because, in *E. cardamomum*, this is a majority compound of its essential oils.

The compounds identified as major in the aqueous extract of *C. longa* have been previously reported [37]. Meanwhile, the anti-inflammatory activity exhibited by this plant has been generally associated with curcuminoids, specifically curcumin [38]. However, curcuminoids were not identified in our aqueous extract of *C. longa*; instead, turmerones (AR, *α*, and *β*) were identified as major compounds, which have not received extensive investigation in regards to their anti-inflammatory activity. In this regard, there have been some reports of the anti-inflammatory properties of turmeric essential oils [29], where Ar-turmerone and *β*-sesquiphellandrene have been reported as the main components considered responsible for such activity [39], specifically the ability of Ar-turmerone to inhibit the production of INF-*γ* and IL-2 [40]. Thus, it is possible to demonstrate the ability to induce the expression of anti-inflammatory interleukins and inhibition of pro-inflammatory interleukins, justifying the use of extracts from *C. longa* or *E. cardamomum* as a treatment for inflammatory conditions.

Different research demonstrated the close relationship between food and health, highlighting the benefits of vitamins, minerals, fatty acids, probiotics, prebiotics, or phytochemicals in fighting various diseases [41]. Among them, the role of substances of plant origin, such as carotenoids, phenolic compounds, alkaloids, nitrogen, and organosulfur compounds, should be marked for their demonstrated influence on the immune system [42].

Some of the immune processes that take place when infections occur are mainly the excessive production of proinflammatory cytokines, including IL-6, IFN-*γ*, IL-1b, and TNF-*α*. Such expression has been closely related to apoptosis-inducing inflammatory processes in animal models [25]. Neutrophil infiltration can lead to damage stimulated by oxidative stress, which increases inflammation and activation of nuclear factor-kappa beta (NF-kB)-dependent pathways. NF-kB induces the production of cyclooxygenase 2 (COX-2), which promotes the production of prostaglandins and other inflammatory agents of the metabolic pathway involved in inflammatory diseases [43]. The presence of the enzyme inducible nitric oxide synthase (iNOS) and COX-2 induces damage associated with excessive production of reactive oxygen species (ROS) and suppression of the antioxidative and defense system [44]. The regulation of iNOS for nitric oxide production will determine whether this will be associated with damage or repair [24]. Some reports have indicated that, in the case of inflammatory processes, chronic inflammation is largely due to uncontrolled production of nitric oxide (NO) by mucosal cells—in this situation nitric oxide may not be properly regulated by iNOS [45]. The arginine pathway plays an important role in tissue repair as L-arginine is converted to an amino acid after tissue injury. Some studies have indicated that arginine increases collagen deposition and tear strength (effects that contribute to epithelial repair and arginase repair functions associated with inflammatory processes); this enzyme inhibits nitric oxide synthesis, resulting in limited intracellular L-arginine supply to produce reactive nitrogen species (RNS) [46], decreasing mucosal damage, such as oral.

In a study conducted in hamsters in which an inflammatory process was induced through local lacerations and a potent inflammatory agent such as 5-fluorouracil, it was observed that daily topical treatment with extracts of *Calendula officinalis* on days 12 to 17 after the inflammatory induction reduced the clinical severity of the disease in a concentration-dependent manner, compared to animals treated only with the vehicle—this is another example of how natural extracts are a good option in the management of lesions [47]. Another study demonstrated the anti-inflammatory effect of chamomile extracts by inhibiting the expression of IL-1β and TNF-α cytokines in an animal model, with data like those observed in our research; however, they could also prove it at the histopathological level, supporting the idea of the use of plant extracts as possible natural candidates in the management of inflammatory processes [48]. In another study, the anti-inflammatory effect of cardamom extracts was demonstrated by inhibiting the action of macrophages producing proinflammatory cytokines such as Il-1*β*, TNF-*α*, and Il-8 through the effect of LPS from *Actinobacillus actinomycetemcomitans* [13]. On the other hand, using a mouse peritoneal macrophage model, the ability of an aqueous extract of cardamom to attenuate IL-6 and TNF-*α* secretion was demonstrated [49]. It has been suggested that the anti-inflammatory activity of cardamom extracts reported by other authors is related to the presence of phytochemical agents in high amounts, in this case to 1,8-cineole (eucalyptol), since this compound has also been shown to attenuate LPS-induced inflammatory signaling pathways in the lung by alveolar macrophages [50].

The present investigation found a positive response of almost 6 relative units of expression for IL-6. The technique was proved valid by positive controls in alpaca enterocytes, which indicated its scarce participation as an inducer of the acute phase of inflammation where leukocytes participate—IL-6 has anti-inflammatory as well as pro-inflammatory effects by regulating the expression of other pro-inflammatory cytokines and, in addition, it induces the synthesis of glucocorticoids [51]. Quantification of cytokine and receptor mRNA was performed by RT-qPCR, from which the results showed positive responses, specifically of *C. longa* concerning IL-10, which is the interleukin responsible for regulating and decreasing the inflammatory response produced by dendritic cells and macrophages, and also for reducing the adaptive responses of CD4 T cells and IL-4, which is a cytokine that acts as an anti-inflammatory by blocking the synthesis of IL-1, TNF-*α*, IL-6, and macrophage inflammatory protein [52]. The proinflammatory activity of INF-*γ*, which is produced in T cells and activated NK cells, was also observed. It potentiates the effects of type I interferons released by Th1, which regulates leukocytes at the site of infection, giving rise to inflammation [53]. As for IL-6, the results dictate a positive response since this interleukin shows anti-inflammatory as well as pro-inflammatory activity [54]. In addition, the extract also influenced the proinflammatory cytokine TNF-*α*, which also leads to the recruitment of inflammatory cells.

Several in vitro studies in human cell lines have demonstrated the cytotoxic capacity of several natural extracts [55,56] such as curcuminoids [16]. Different components of *Zingiberaceae* plants can suppress the activity of some common mutagens and carcinogens in various cell types in both in vitro and in vivo studies. The cytotoxic effects of methanol extracts and their fractions (hexane, ethyl acetate, and water) of *Alpinia mutica* rhizomes against six human carcinoma cell lines (KB, MCF7, A549, Caski, HCT116, HT29) and the non-human fibroblast cell line (MRC 5) are currently known as a result of an in vitro cytotoxicity assay [57]. In two studies on colon and prostate cancer, curcumin from *C. longa* inhibited cell proliferation and tumor growth [58]. In addition, curcumin is a nonmutagenic and nongenotoxic agent [15]. Although the cytotoxic activity of curcuminoids is known, in this research, promising results were observed in terms of anti-inflammatory activity, but no cytotoxic activity was observed in HeLa, J774A.1, Vero E6, and peritoneal cell lines, as the EC_50_ values found were higher than 200 µg/mL and EC_90_ values were higher than 400 µg/mL, respectively, for all assays evaluated (Table 6).

Currently, there is a growing interest in both industry and scientific research on spices and aromatic herbs due to their strong antioxidant powers and antimicrobial properties—both natural and synthetic antioxidants are currently being used. In some of these studies, microbicidal activity against *Streptococcus mutans* was observed with an increase of nitric oxide in RAW 264.7 macrophages stimulated with extracts of *Rosmarinus officinalis*, *Thymus vulgaris*, and *C. longa* [59]. Another study showed that curcumin at a dose of 0.5 ppm induced polarization to an M2 phenotype or alternating activation with anti-inflammatory characteristics in peritoneal macrophages, although at higher doses this phenotype was lost and they observed increased expression at the level of Arg-1 transcripts, which is a competitor of L-Arginine against iNOS enzymes [60]. When we stimulated macrophages with LPS in the presence of the extracts, nitrite production decreased, in addition to the fact that the extract alone induced little nitrite production without stimulation in both aqueous extracts. These data suggest the anti-inflammatory effect of the extracts alone or in combination with LPS (Figure 3). At the moment, the modulatory effects of turmeronole A and B (plant components of the *C. longa*) on RAW 264.7 cells in inflammatory processes are known—they significantly inhibited LPS-induced prostaglandin E (PgE) and NO production, as well as the expression of iNOS and the gene encoding PgE. In addition, turmeronols significantly inhibited the overexpression of transcripts encoding IL-1*β*, IL-6, and TNF-*α* at the mRNA and protein level induced by LPS [61].

In addition, several investigations carried out with the main components in plants of this family have demonstrated their antioxidant capacity against the DPPH radical in comparison with Trolox and ascorbic acid—among the most important ones are *α*-turmerone, *β*-turmerone, and Ar-turmerone [62]. In an investigation to evaluate the hepatoprotective effect of an extract of *Amomum cardamomum* on carbon-tetrachloride-induced liver damage through antioxidant activity in rats, it was found to possess significant hepatoprotective activity on acute liver injury, which could be derived from its antioxidant properties and the decrease of liver cytochrome P450 [63]. In general, it can be stated that the pharmaceutical properties of plants of the *Zingiberaceae* family are related to their chemical composition; this is mainly due to the presence of phenolic compounds and other biologically active constituents [11]. In addition to anticancer, antioxidant, and free radical scavenging effects, these plants possess the capacity to indirectly increase glutathione levels, thus aiding in the hepatic detoxification of mutagens and carcinogens and inhibiting the formation of nitrosamines [58].

We think that the results presented in this manuscript may be sufficient to infer the anti-inflammatory effects of the extracts in question, given that several investigations have provided evidence such as those presented in this document—in the case of *E. cardamomum*, some authors have mentioned the anti-inflammatory properties [13,49] and for *C. longa*, some authors described these same effects through the suppression of NO and COX [64,65].

## 5. Conclusions

Under the experimental conditions analyzed and based on the results obtained in the tests performed in this research, the anti-inflammatory effects of *E. cardamomum* and *C. longa* were evidenced. The aqueous extracts of cardamom and turmeric showed elevated expression of interleukins, suggesting their possible anti-inflammatory or immunomodulatory action. As for the cytotoxic action, a dose–response relationship was observed, since as the concentration increased, the cytotoxic activity increased. The concentrations evaluated (from 3.125 to 200 µg/mL) showed no activity against HeLa, J774A.1 and Vero E6 cell lines. In future research, we expect to continue with more studies to identify the molecular mechanisms of action that favors the use of these plants as complementary alternatives in inflammatory processes in order to develop alternative or adjuvant therapies, as well as safe applications of cardamom and turmeric, which could bring several benefits, such as cost reduction compared to existing products, in addition to contributing to the ethno-pharmacological development for the safe use of folk traditional plants.

## Figures and Tables

**Table 1:** Concentrations of aqueous extracts evaluated.

Aqueous Extract	Concentration (µg/mL)
*E. cardamomum*	100
*C. longa*	70

**Table 2:** Sequences of oligonucleotides used for RT-qPCR.

Gene	Genebank ID	Oligonucleotides
**IL-4**	NM_021283.2	F: 5′ TTG AGA GAG ATC ATC GGC ATT T -3′ R: 5′ CTC ACT CTC TGT GGT GTT CTT C -3′
**IL-6**	NM_031168.2	F: 5′ CTT CCA TCC AGT TGC CTT CT -3′ R: 5′ CTC CGA CTT GTG AAG TGG TAT AG -3′
**IL-10**	NM_010548.2	F: 5′ TTG AAT TCC CTG GGT GAG AAG -3′ R: 5′ TCC ACT GCC TTG CTC TTA TTT -3′
**Gpd1**	NM_010271.3	F: 5′ CCT ACT GCT GAC CTT TCT TCT C -3′ R: 5′ GCC CTG AGG ACG ATA AAC TAT AA -3′
**TNF-α**	NM_013693.3	F: 5′ TTG TCT ACT CCC AGG TTC TCT -3′ R: 5′ GAG GTT GAC TTT CTC CTG GTA TG -3′
***β*** **-actin**	NM_07393.5	F: 5′- GAG GTA TCC TGA CCC TGA AGT A -3′ R: 5′- CAC ACG CAG CTC ATT GTA GA -3′

**F:** Forward; **R:** Reverse.

**Table 3:** Phytochemical tests.

Test	Chemical Groups	*E. cardamomum*	*C. longa*
Lieberman Burchard	Sterols, triterpenes	+	+
Shinoda	Flavonoids	+	+
Baljet	Sesquiterpene lactones	+	+
Sulfuric acid	Quinones	−	−
Ferric chloride	Tannins	+	−
Yield %		5.64	9.83

**+** Positive reaction; **−** negative reaction.

**Table 4:** Main chemical composition of aqueous extracts of two *Zingiberaceae* plants identified by GC-MS/MS.

Plant Extract	Chemical Compound	R.T.	P.A. %	M.W. (g/mol)
*E. cadamomum* (Cardamom)	*α*-terpinyl acetate	10.27	54.46	196.29
	2-((1R,4R)-4-hydroxy-4-methylcyclohex-2-enyl) propan-2-yl acetate	11.31	3.55	212.28
	9-hexacosene	26.96	3.52	364.69
	*γ*-sitoesterol	29.93	3.38	414.71
	Heneicosane	24.73	3.04	296.57
	8-acetoxycarvotanacetone	12.93	2.57	210.27
	Geranyl oleate	27.24	2.28	418.70
	(Z)-3,7-dimethylocta-2,6-dien-1-yl palmitate	26.31	2.08	392.70
	Naphthalene, decahydro-4a-methyl-1-methylene-7-(1-methylethenyl)-, [4aR-(4a*α*,7*α*,8a*α*)]-	12.05	2.03	204.35
	*α*-terpineol	8.12	1.95	154.25
	Pentacosane	25.99	1.85	352.68
*C. longa* (Turmeric)	*β*-turmerone	15.51	33.45	218.33
	*α*-turmerone	15.95	21.30	218.33
	Ar-turmerone	15.57	19.85	216.32
	16-kauren-19-yl acetate	27.08	18.72	330.26
	*α*-atlantone	16.80	2.77	218.33
	*β*-sesquiphellandrene	13.84	1.98	204.35
	Benzene	13.31	1.93	78.11

**R.T.**: retention time; **P.A.** %: peak area percent; **M.W.**: molecular weight.

**Table 5:** Cytokines gene expression.

Cytokine	*C. longa*	*E. cardamomum*	LPS
IL-4	5.07 ± 1.6	0.30 ± 0.185	0.00
IL-6	2.21 ± 0.04	1.05 ± 0.012	5.61 ± 0.284
IL-10	1.84 ± 0.87	2.02 ± 0.026	0.90 ± 0.073
TNF-α	1.46 ± 0.97	0.086 ± 0.070	4.59 ± 2.132

**Table 6:** Cell cytotoxicity assay.

				Cell Cytotoxicity Percent (%)				
	*Elettaria cardamomum*				*Curcuma longa*			
Concentration µg/mL	HeLa	J774A.1	Vero E6	Balb/C Peritoneal Cells	HeLa	J774A.1	Vero E6	Balb/C Peritoneal Cells
**3.125**	8.26 ^a^	4.11 ^a^	4.14 ^a^	11.73 ^a^	2.18 ^a^	11.74 ^a^	8.34 ^a^	2.18 ^a^
**6.25**	10.17 ^a^	10.28 ^b^	4.57 ^a^	14.07 ^a^	8.23 ^b^	14.06 ^a^	10.18 ^a^	8.23 ^b^
**12.5**	13.03 ^a^	13.73 ^b^	8.39 ^b^	14.33 ^a^	9.59 ^b^	14.35 ^a^	19.03 ^b^	9.59 ^b^
**25**	13.51 ^a^	22.26 ^c^	12.71 ^b^	16.23 ^a^	13.85 ^b^	16.24 ^a^	21.76 ^b^	13.85 ^b^
**50**	19.47 ^b^	31.31 ^c^	13.04 ^b^	17.60 ^a^	14.63 ^b^	17.62 ^ab^	22.44 ^b^	14.63 ^b^
**100**	20.58 ^b^	37.94 ^c^	19.54 ^c^	20.37 ^ab^	22.16 ^c^	20.39 ^ab^	25.81 ^b^	22.16 ^c^
**200**	21.93 ^b^	45.33 ^d^	35.21 ^d^	27.20 ^b^	22.92 ^c^	27.21 ^b^	26.41 ^b^	22.92 ^c^
**EC_50_ µg/mL**	424.01	237.36	257.51	431.16	351.17	430.96	396.24	362.86
**LL**	372.09	190.79	191.02	287.35	321.20	381.60	349.78	269.66
**UL**	473.84	261.68	301.23	582.74	382.14	480.33	442.71	509.85
**EC_90_ µg/mL**	890.09	423.17	436.37	838.33	721.65	957.64	931.97	705.80
**LL**	811.96	412.91	402.75	690.10	705.80	934.98	905.02	596.56
**UL**	988.53	434.32	478.67	983.15	757.78	982.90	962.03	831.34

The table shows the mean EC_50_ and EC_90_. The lower (LL) and upper limits (UL) are shown. Different letters in each column represent significant differences between the groups analyzed via the Tukey post hoc test.

## Data Availability

The data presented in this research are available on request from the corresponding author.
